# Evaluating MRI response criteria in microsatellite instability-high rectal cancer treated with immune checkpoint inhibitors

**DOI:** 10.1016/j.esmoop.2025.106020

**Published:** 2025-12-29

**Authors:** Q. Vanderbecq, R. Cohen, M. Camus, X. Dray, Y. Parc, T. André, M. Wagner

**Affiliations:** 1 Department of Radiology, AP-HP, Sorbonne, Saint-Antoine Hospital, Paris, France; 2 UMR 7371, Université Sorbonne, CNRS, Inserm U114615, Paris, France; 3 Department of Medical Oncology, AP-HP, Sorbonne, Saint-Antoine Hospital, Paris, France; 4 INSERM UMRS 938, Équipe Instabilité des Microsatellites et Cancer, SIRIC CURAMUS, Centre de recherche Saint Antoine, Paris, France; 5 Centre for Digestive Endoscopy, AP-HP, Sorbonne, Saint-Antoine Hospital, Paris, France; 6 Department of Digestive Surgery, AP-HP, Sorbonne, Saint-Antoine Hospital, Paris, France

**Keywords:** MRI, rectal tumor, immunotherapy

## Abstract

**Background:**

Immune checkpoint inhibitors (ICIs), particularly anti-programmed cell death protein 1 (PD-1) agents, have enabled consideration of non-operative management in patients with microsatellite instability-high (MSI-H) or mismatch repair-deficient (dMMR) rectal cancer. Standard magnetic resonance imaging (MRI) response criteria—developed for chemoradiotherapy—may not accurately reflect treatment effects with ICIs, however.

**Patients and methods:**

We retrospectively analyzed 14 patients with locally advanced MSI-H/dMMR rectal adenocarcinoma treated with neoadjuvant anti-PD-1 monotherapy. All patients achieved a complete pathological response. MRI assessment included T2-weighted, diffusion-weighted, and contrast-enhanced sequences. Treatment response was defined as complete pathological response by surgical pathology (proctectomy), or by endoscopic and biopsy-confirmed complete remission (watch-and-wait strategy).

**Results:**

MRI showed distinct post-treatment patterns: split scar sign (*n* = 2), fibrotic response (*n* = 5), lesion disappearance (*n* = 1), and residual intermediate T2 signal (*n* = 6). All mucinous tumors retained T2-hyperintense residues. In select cases, fibrotic response was beyond 3 months. In two surgical cases, histological analysis revealed residual mucinous material without viable tumor cells.

**Conclusions:**

MRI response patterns after anti-PD-1 monotherapy in MSI-H/dMMR rectal cancer differ from those observed after radiochemotherapy, suggesting the need to adapt current imaging criteria for accurate response assessment and informed surgical decision making. Persistent MRI abnormalities are frequent despite complete pathological response, and warrant cautious interpretation in the ICI setting.

## Highlights

• MRI complete remission was 36% (5/14), rising to 50% (5/10) when mucinous tumors were excluded.
• All mucinous tumors (4/4) retained T2-hyperintense signals despite the absence of viable tumor cells.
• In select cases, a delayed fibrotic response was observed for >3 months.
• MRI response patterns in MSI-H/dMMR rectal cancer differ between anti-PD-1 monothearpy and radiochemotherapy.

## Introduction

Immune checkpoints inhibitors (ICIs) have revolutionized the treatment of mismatch repair-deficient [microsatellite instability-high/deficient mismatch repair (MSI-H/dMMR)] metastatic colorectal cancers,^1^, ^2^, ^3^ demonstrating durable responses and survival benefits even in chemo-refractory settings. As a result, ICIs have become the standard of care for patients with MSI-H/dMMR metastatic colorectal disease.^1^, ^2^, ^3^ In rectal cancer, the efficacy of ICIs has been further highlighted in a recent phase II trial, where single-agent programmed cell death protein 1 (PD-1) dostarlimab led to a 100% clinical complete response rate in all 49 patients who completed treatment and were managed non-operatively.^4^ These unprecedented outcomes challenge traditional treatment paradigms by eliminating the need for chemotherapy, radiotherapy, and surgery in selected patients. High-resolution magnetic resonance imaging (MRI) and other advanced imaging modalities now play a pivotal role in identifying complete responders and guiding watch-and-wait strategies in rectal cancer.^5^ Accurate imaging-based response assessment ensures that patients with robust tumor regression can safely avoid surgery without compromising oncological outcomes.^6^ Currently, post-neoadjuvant chemoradiotherapy response evaluation in rectal cancer is based on the MRI tumor regression grade (mrTRG), using high-resolution T2-weighted MRI and diffusion-weighted imaging.^7^ Immune-related tumor response may differ fundamentally from radiation-induced changes, however. Immune-related changes such as delayed responses, persistent mucinous or fibrotic signal abnormalities without a viable tumor, and phenomena such as pseudo-progression have been increasingly reported after ICI treatment.^8^, ^9^, ^10^ These immune-related patterns may not be reliably captured using conventional mrTRG criteria, which were developed and validated specifically for assessing tumor regression after chemoradiation. Can conventional MRI response criteria, developed for monitoring chemoradiotherapy, therefore reliably evaluate follow-up response in the setting of treatment with ICIs?

## Materials and methods

All consecutive patients diagnosed with locally advanced MSI-H/dMMR rectal cancer treated with anti-PD-1 monotherapy between 2022 and 2025 at Saint-Antoine Hospital, France were included in the prospective ImmunoMSI cohort approved by the ethics committee (N°2020 – CER 2020-6). We retrospectively reviewed, with institutional review board approval, the clinical and imaging outcomes of 16 patients [10 women, 6 men; median age: 59 years (range 25-73 years)]. Patients were enrolled in ICI(s) trials or were treated under a compassionate use program. Baseline thoraco-abdominopelvic computed tomography evaluations were carried out for all patients for eliminating metastatic disease. All patients underwent MSI-H/dMMR screening via polymerase chain reaction and/or immunohistochemistry.

The MRI protocol included multiplanar T2-weighted imaging, diffusion-weighted imaging, and intravenous contrast-enhanced imaging [carried out in five patients (36%)] at baseline and during follow-up. All images were independently reviewed by two abdominal radiologists with 4 and 12 years of experience, who were blinded to the endoscopic findings. In cases of disagreement, a final consensus interpretation was reached. Response was defined either by histopathological complete tumor clearance (pathological complete response) in the surgical resection specimen or by endoscopic visualization of complete remission with biopsy-confirmed absence of tumor cells (*n* = 12). Follow-up MRI was carried out every 3 months including MRI within 1 month of pathological complete response in 12 patients (86%), and within 3 months in all evaluated patients (*n* = 14). Descriptive statistics were computed using Python. Continuous variables are reported as medians with ranges, and categorical variables as counts and percentages.

## Results

Two patients were excluded from the final analysis: one due to the absence of a pre-response MRI carried out within 3 months of clinical evaluation (histopathology after resection revealed residual pT1 disease), and one due to non-interpretable baseline imaging of insufficient quality. At baseline, all patients (*n* = 14) presented with locally advanced MSI-H/dMMR rectal cancer, classified as either T3 (*n* = 10, 71%) or T4 (*n* = 4, 29%), with undetermined nodal status (Nx). All cases demonstrated visible mesorectal lymph nodes on MRI. The tumor location was the lower rectum (*n* = 6; 43%), middle rectum (*n* = 5; 36%), or upper rectum (*n* = 3; 21%). The median baseline cranio-caudal length measured by MRI was 46.5 mm (range 28-86 mm), and median mural thickness was 13 mm (range 9-31 mm). Four patients (29%) initially exhibited at least a partially mucinous component at baseline, characterized by T2-hyperintense, well-demarcated regions without diffusion restriction, consistent with intratumoral mucin pools. Table 1 summarizes patient and tumor characteristics.

**Table 1:** Patient and tumor baseline characteristics and patient follow-up data

	Value
Enrollment	
Total patients enrolled, *n*	16
Excluded from final MRI analysis, *n* (%)	2 (12)
Patients analyzed, *n*	14
Patient characteristics	
Sex	5 female/9 male
Age (years), median (range)	59 (25-73)
Management after response, *n* (%)	Surgery: 2 (14) Watch-and-wait: 12 (86)
Median clinical follow-up, months (range)	17.5 (9-36)
Lynch syndrome, *n* (%)	5 (36)
Immune checkpoint therapy, *n* (%)	Dostarlimab: 9 (74) Pembrolizumab: 5 (26)
Tumor characteristics, *n* (%)	
Baseline cT category	T3: 10 (71) T4: 4 (29)
Baseline nodal status (MRI)	N0: 0 N1-N2: 14 (100)
Tumor location	Lower: 6 (43) Mid: 5 (36) Upper: 3 (21)
MRI at baseline	
Cranio-caudal length, mm (range)	46.5 (28-86)
Mural thickness, mm (range)	13 (9-31)
Partially or wholly mucinous, *n* (%)	4 (29)
Contrast-enhanced MRI carried out, *n* (%)	5 (36)
MRI at complete pathological response	
MRI complete response, *n* (%)	5 (36)
Baseline cranio-caudal length, mm (range)	24 (0-45)
Cranio-caudal length shrinkage, median (range), %	45 (21-100)
Mural thickness, mm (range)	7.5 (0-14)
Mural thickness shrinkage, median (range), %	37 (0-100)
Mesorectal nodes present, *n* (%)	14 (100)

Complete pathological response was defined by surgical pathology report showing complete pathological response on surgical specimens (*n* = 2), or by endoscopic and biopsy-confirmed complete remission (*n* = 12).

cT, clinical primary tumor; MRI, magnetic resonance imaging.

**Figure 1:**
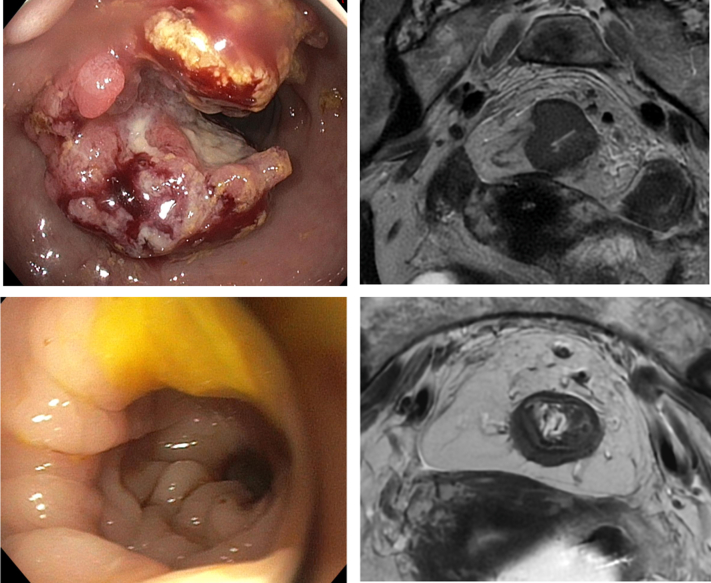
**Lesion evolution with endoscopic correlation.** Top row: baseline axial T2-weighted MRI of a high rectal lesion, with corresponding endoscopic image. Bottom row: follow-up MRI demonstrating a split scar sign pattern, indicative of treatment response.

**Figure 2:**
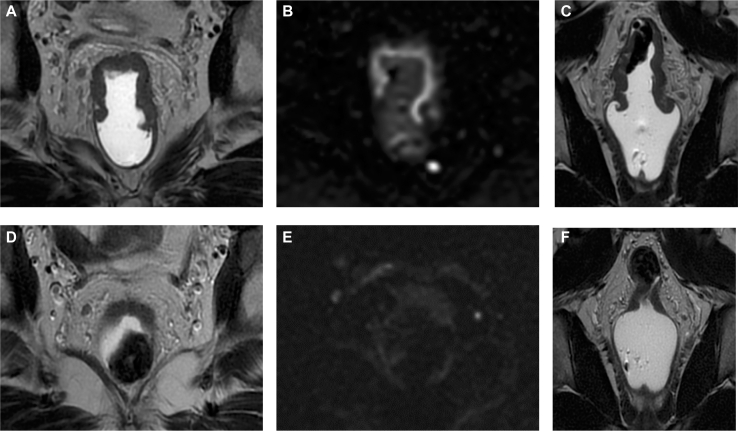
**Representative lesion of the mid rectum.** Top row (A-C): Baseline MRI. (A) Axial T2-weighted image showing an intermediate signal intensity lesion, (B) Diffusion-weighted image (*b* = 1500) demonstrating rim-restricted hyperintensity, (C) Coronal T2-weighted image. Bottom row (D-F): Post-treatment MRI showing persistent mural thickening without fibrotic low signal. (D) Axial T2-weighted image with unchanged intermediate signal, (E) Axial diffusion-weighted imaging demonstrating resolution of the hyperintensity, (F) Coronal T2-weighted image. Despite these persistent imaging findings, endoscopy and biopsy revealed no viable residual tumor cells, illustrating a discordance between MRI and radiological–pathological findings after ICI therapy.

The median follow-up period was 17.5 months (range 9-36 months). MRI response patterns following single-agent anti-PD-1 differed from those typically seen after chemoradiation. Specifically, two patients (14%) demonstrated the classic split scar sign (Figure 1); five patients (36%) showed at least partial fibrotic remodeling, including two classified as mrTRG2; and one patient (7%) showed complete radiological disappearance of the lesion. Six patients (43%) demonstrated isolated lesion shrinkage with persistent intermediate T2 signal intensity. This pattern was observed in 3 of 10 patients (30%) when excluding predominantly mucinous tumors (Figure 2). Post-treatment, the median mural thickness decreased to 7.5 mm (range 2-14 mm), corresponding to a 42% median reduction from baseline. Similarly, the median craniocaudal length decreased to 26 mm (range 0-44 mm), reflecting a 44% median reduction. Notably, all four patients (29%) with initially mucinous or partially mucinous tumors retained persistent T2-hyperintense mucinous residues after treatment.

Among patients who underwent early MRI evaluation (*n* = 6, 43% of the cohort) within <3 months of completing neoadjuvant therapy but did not demonstrate definitive MRI response features (i.e. absence of mrTRG1/2 or the split scar sign), a delayed MRI assessment was carried out at a median interval of 3 months (range 3-8 months). Of these six patients, three (50%), all with initially mucinous tumors, demonstrated a delayed peripheral fibrotic response consistent with mrTRG2. Two patients (33%) showed no significant morphological change and remained classified as mrTRG3/4, with persistent intermediate T2 signal and no clear fibrotic remodeling. One patient (17%) demonstrated the emergence of a delayed split scar sign, indicating ongoing tissue remodeling and potential delayed response. One additional patient from this subgroup lacked follow-up MRI and was excluded from the delayed imaging analysis.

Two patients underwent surgical resection due to incomplete MRI response, despite complete endoscopic response. As these were among the first patients treated with anti-PD-1, the presence of a persistent visible lesion on MRI and multiple mesorectal lymph nodes raised concern, prompting surgery. Histological analysis in both cases revealed residual mucinous material, but no viable tumor cells.

In the remaining 12 patients who were managed non-operatively, endoscopic evaluations revealed inflammatory or scarred mucosa. Targeted biopsies confirmed the presence of inflammatory or granulation tissue without any residual tumor cells.

## Discussion

**Figure 3:**
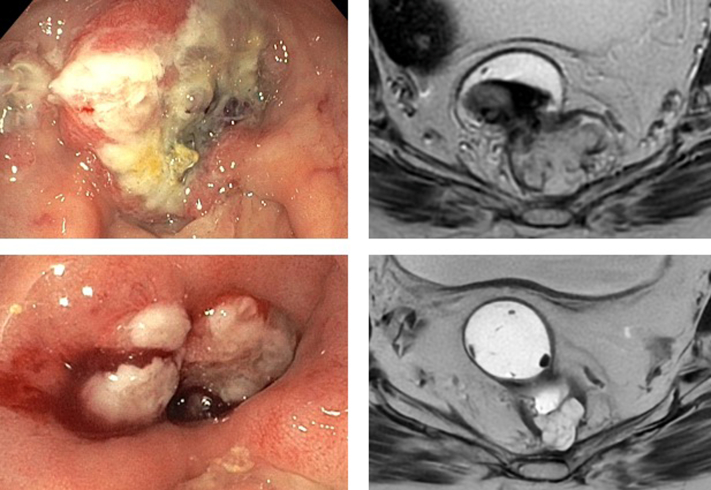
**Mucinous lesion with endoscopic correlation.** Top row: baseline axial T2-weighted MRI and corresponding endoscopic view of a mucinous rectal tumor. Bottom row: follow-up MRI showing persistent mucinous signal; endoscopy revealed a small area of exuberant granulation tissue at the previous tumor site.

Our findings highlight distinct MRI phenotypes in MSI-H/dMMR rectal cancer after anti-PD-1 monotherapy, including persistent mucinous or intermediate T2 signal residues rather than the fibrotic T2 hypointensity seen after chemoradiation. Recognizing these MRI features is essential for accurate response evaluation and staging, particularly to avoid unnecessary proctectomy in the case of complete pathological response. In patients who underwent follow-up MRI >3 months after achieving clinical pathological response, several initially indeterminate lesions evolved into clearly fibrotic tissues or demonstrated a delayed split scar sign. This supports the value of flexible and extended surveillance in clarifying ambiguous MRI patterns before labelling residual disease. Although no recurrence was observed during the median follow-up period of 17.5 months, longer surveillance is warranted for all patients to confirm the durability of complete responses, particularly in those with mucinous tumors, where residual mucin pools may persist and could harbor delayed recurrence (Figure 3).

These results align with a recent dedicated MRI-pathology correlation study in MSI-H/dMMR rectal cancer, which reported pseudo-residue and pseudo-progression in 77% and 23% of tumors, respectively, after PD-1 blockade, with patterns not observed after chemoradiation.^11^ Moreover, conventional MRI sequences cannot reliably distinguish acellular from cellular mucin, leading to high false-positive rates for persistent disease in mucinous subtypes.^12^ These findings are consistent with our results and reinforce the conclusion that mrTRG cut-offs derived from chemoradiotherapy are overly restrictive when applied to immunotherapy-treated tumors. Two mechanisms likely underline these discrepancies: (1) dense lymphoid stroma and collagenous scar tissue, hallmarks of immune-mediated regression, may mimic viable tumor on T2-weighted MRI; and (2) mucin itself remains hyperintense regardless of its cellularity, complicating interpretation. Persistent mucin-related T2 hyperintensity therefore limits MRI response assessment across treatment modalities, whether after immunotherapy or chemoradiotherapy, since conventional MRI sequences cannot reliably distinguish acellular from cellular mucin. Importantly, our experience suggests that delayed MRI, carried out at least 3 months after clinical response, can clarify ambiguous cases, converting several indeterminate patterns into definitive fibrotic or split scar appearances. This suggests that extending the imaging interval may reduce false classification of non-response and should be considered in tailoring response criteria for ICI blockade. Timing must be factored across all modalities of response assessment, MRI, endoscopy, or biopsy. In the study by Cercek et al.,^4^ which included MSI-H/dMMR tumors treated with dostarlimab (not limited to rectal cancer), the median time to complete response was 6.2 months on imaging (range 2.6-10.0 months), 6.1 months on endoscopy (range 1.2-9.8 months), and only 1.5 months on biopsy (range 1.1-9.8 months).^4^ These data support a stepwise evaluation strategy discussed within a multidisciplinary team and, except in cases of clear progression, recommended at least 6 months of surveillance before deciding between a watch-and-wait approach and surgical resection.

Limitations of this study include its retrospective, single-center design, small sample size, and the fact that all patients achieved complete histological response at endoscopy or on pathological analysis after proctectomy, limiting generalizability. Imaging was interpreted by expert radiologists in a specialized setting, which may affect reproducibility in broader clinical practice. Most patients were managed non-operatively, and complete response was confirmed by endoscopy and target biopsy rather than whole-specimen histology, introducing a potential risk of sampling error. Finally, advanced imaging techniques such as Fibroblast Activation Protein Inhibitor Positron Emission Tomography,^13^ were not included, preventing evaluation of novel imaging biomarkers and response patterns.

Further prospective studies are needed to validate these MRI response patterns and refine imaging response criteria tailored specifically to MSI-H/dMMR rectal adenocarcinoma. This would support more accurate, evidence-based decisions regarding the safely of non-operative management. Based on our findings, careful surveillance remains essential. In cases showing residual mucinous components, incomplete fibrotic remodeling, or persistent mesorecta nodes, we recommend repeating MRI and endoscopic assessments every 3 months, maintaining a non-operative watch-and-wait approach as long as no progression is observed.
